# Changes to Corporate Restructuring Laws in the Czech Republic During the Covid-19 Pandemic

**DOI:** 10.1007/s40804-023-00273-7

**Published:** 2023-03-13

**Authors:** Jan Lasák

**Affiliations:** 1 Commercial Law Department, Masaryk University, Brno, Czech Republic; 2 Partner, Kocián Šolc Balaštík, advokatni kancelar, s.r.o., Prague, Czech Republic

**Keywords:** Covid-19 pandemic, Restructuring laws, Bail-in, Bail-out, Extraordinary moratorium, Corporate governanc

## Abstract

In the course of the Covid-19 global pandemic, corporate restructuring laws worldwide underwent a stress test as the financial health of companies significantly deteriorated. In response to public demand, legislators in the Czech Republic adopted various bail-out programmes and bail-in measures in order to provide debtors who ran into temporary problems in connection with the Covid-19 pandemic with additional protection against individual creditors to help solve their financial troubles. This paper outlines the various bail-in and bail-out measures that were introduced during the critical phase of the pandemic in 2020–2021 and analyses the extraordinary measures that were adopted in the Czech Republic. After the pandemic, once the temporary emergency measures were lifted, the number of insolvency petitions and proceedings surprisingly decreased in comparison to previous years. This may suggest that financial aid programmes in the Czech Republic may even have been too generous. While public debt in the Czech Republic increased dramatically during the pandemic as a result of the governmental bail-out/financial aid programmes, the ‘helicopter money’ contributed significantly to accelerating the increase in the country’s inflation rate.

## Introduction

In many respects, the global pandemic of the novel coronavirus known as SARS CoV-2 posed an unusual challenge to legal systems across continents, whether in terms of restricting fundamental human rights and freedoms or prohibiting/restricting certain forms of business where human contact is the norm. In the Czech Republic, the government and the Ministry of Health, in response to the spread of the coronavirus, issued a number of emergency measures restricting, in particular, the free movement of persons in the Czech territory.^1^

The Czech Ministry of Health took emergency measures against the epidemic and the danger of its occurrence, for the first time on 6 March 2020, whereby anyone returning from Italy was ordered to undergo a mandatory 14-day quarantine. Due to the rapid local spread of the disease, the Regional Health Office of the Olomouc Region subsequently adopted another emergency measure (lockdown), which led to a fundamental reduction of everyday life in selected municipalities in the vicinity of Litovel and Uničov in the Olomouc Region. This completely isolated the area from the outside world and prohibited the free movement of local residents. In view of the deteriorating situation, on 12 March 2020, the Czech government declared a state of emergency for the entire Czech Republic and adopted radical emergency measures under the Emergency Management Act,^2^ which resulted in a fundamental restriction of the free movement and assembly of persons in the Czech Republic.

The pandemic and the subsequently introduced emergency measures significantly impacted on various spheres of everyday life. For precautionary reasons, people began to stay in their homes and stopped meeting each other unless absolutely necessary (social distancing). The operation of the Czech Postal Service was limited, and some of its branches were completely closed, and shopping centres were generally closed as well. Given the restrictions on the free movement of persons, the ban on people to assemble in the same place, and the quarantine measures that were applied in the Czech Republic, many legal (business) entities found it difficult or even impossible to hold meetings, especially meetings of their supreme bodies (such as general meetings of shareholders of limited liability companies or joint-stock companies).^3^

The emergency measures introduced because of the pandemic had a major impact on businesses in many sectors, with not only small and medium-sized businesses (SMEs) but also large companies running into financial problems, such as ČSA, Smartwings airlines and FIRO-tour (one of the largest travel agencies in the Czech Republic). A number of industries (especially restaurants, hotels and retail shops) came to a complete standstill as these businesses experienced significant reductions in their revenue or stopped being economically viable at all. Calls for a swift response from the government started coming from all parts of the economy in terms of (i) financial support and (ii) changes to the standard legal framework for corporate governance and insolvency/restructuring laws.

This paper dives into that particular moment and is structured as follows. In Sect. 2, I begin with an outline of the various bail-in and bail-out measures that were introduced during the critical phase of the pandemic in 2020–2021. In addition to various forms of financial support introduced by the government, the latter considered the standard regulatory framework to be institutionally insufficient to adequately respond to the ongoing crisis. In response to public demand, a set of temporary regulatory measures were adopted in the area of company restructuring laws and corporate governance, which are discussed in the subsequent sections. As the protective measures (both the financial support and the regulatory changes to the law) were only temporary, I conclude by discussing the development of what happened when these measures were lifted.

## Financial Support (Bail-ins and Bail-outs)

In a ‘healthy’ economy, restructuring and insolvency law helps to ‘cleanse’ the business environment of failed businesses and serves to efficiently allocate assets in the economy. However, if there is a ‘black swan’ type of event, restructuring and insolvency law may not properly fulfil this purpose, notably because too many assets may come onto the market at the same time for which there is no corresponding demand, which can have a significant impact on the assets’ sale price below its fundamental value and, accordingly, can have a detrimental effect on both debtors and their creditors (risk of fire-sale outcomes).^4^ In addition, financial problems of a large part of the economy can also pose a systemic kind of risk which may threaten society as a whole. In order to face the ongoing crisis, the Czech government took a variety of steps to mitigate the impact of the Covid-19 pandemic. In addition to several bail-out programmes that were implemented on the basis of the EU Temporary Framework for State Aid Measures to Support the Economy in the Current Covid-19 Outbreak,^5^ the Czech government also introduced various bail-in measures.^6^

### Bail-in Measures

Inspired by German regulation,^7^ protection for businesses with commercial leases was introduced that prohibited the leases from being terminated for non-payment during the pandemic.^8^ Tenants were in no way released from their obligation to pay rent; nor could they demand any reduction in the amount of rent. It merely involved a deferral of payments, and only of rental payments, not of payments for services. Tenants were obliged to pay the rent due by 31 December 2020 as the postponement of rental payments was not, in contrast to other emergency measures, prolonged into 2021. Instead, governmental bail-outs started to include substantial contributions to the payment of rent for businesses (Covid-Rents programme^9^) that had to temporarily close their retail and customer service outlets due to the emergency measures while still being required to continue paying rent under their lease agreements.

As part of the financial bail-in support, special Act No. 177/2020 Coll. on certain pandemic-related loan repayment measures was introduced as another piece of legislation that came into effect during the pandemic period (on 17 April 2020). This Act responded to the (potential) temporary loss of the ability of borrowers to repay loans due to the Covid-19 pandemic. The underlying principle of this regulation was the ability to temporarily suspend (without penalty) the repayment of selected loans in respect of mortgages and other similar loans arranged in relation to real estate before 26 March 2020 and all other loans (subject to statutory exceptions) arranged and drawn down before 26 March 2020 (including investment and other loans drawn down by businesses).^10^

Suspension of loan repayments under the Act did not occur automatically, but only on the basis of a unilateral notification by the borrower to the lender that it intended to use the grace period. Under the Act, the protection period started from the first day of the calendar month following the day on which the borrower delivered the notification to the lender, until 31 October 2020. Utilising the protection period did not result in any waiver of the debt, but only postponed the time for fulfilling the monetary debts (individual instalments) under the relevant loan agreement. Accordingly, the repayment period of the loan was extended. If the borrower was not a consumer, it had to pay at least the agreed interest or related charges during the protection period.

Furthermore, a special bail-in regulation concerning default in the payment of monetary debts was introduced.^11^ If the debtor proved that timely repayment of a cash debt had been thwarted or substantially hindered by the emergency restrictions, the creditor could merely request the debtor to pay penalties up to the statutory default interest rate (Czech National Bank repo rate + 8% points). A penalty was understood to be any negative pecuniary consequence of the debtor’s default (in particular, default interest, contractual fines, penalties or other agreed price premiums).^12^ The limitation on penalties applied primarily to financial debts which had to be repaid during the pandemic (i.e., where no statutory postponement of loan repayment under Act No. 177/2020, as described above, applied). The adjustment was applicable until 30 June 2020, i.e., it applied to the debtor’s default in the period from 12 March 2020 (when a state of emergency was declared in the Czech Republic) to 30 June 2020, but not to obligations arising after Lex Covid (defined below) came into force (i.e., after 24 April 2020). The provision was of a mandatory nature, i.e., different contractual arrangements were disregarded unless they provided lower penalties than those permissible under the Lex Covid regulation.

### Bail-out Measures

The most significant measure taken by the Czech government, however, was the financial (bail-out) support offered to moderate the negative economic consequences of the Covid-19 pandemic. It took the following forms:^13^

- *direct bail-out support* in the form of large non-repayable subsidies (compensation) provided by various government agencies. The backbone of the public subsidies programme was (i) a so-called ‘compensation bonus’ to compensate for turnover losses caused by the regulatory measures introduced to prevent the spread of the coronavirus (which prohibited, for example, the assembly of a large number of people);^14^ (ii) a contribution to compensate for the costs of paying wages (coined ‘Antivirus’); and (iii) a contribution to the payment of rent for businesses that had to temporarily close their retail and customer service outlets due to the emergency measures while still being required to continue paying rent under their lease agreements. Special forms of direct support included, for example, aid to businesses to compensate for the costs and operating losses caused by the obligation to close Advent and Christmas markets in 2020, to businesses which could not operate their restaurants and/or accommodation establishments, or to businesses organising sporting events.
- *Indirect bail-out support*, in the form of both (i) a so-called ‘liberation package’ (involving deferral of tax obligations for all businesses whose activities were restricted or prohibited by the emergency measures introduced),^15^ and (ii) loans or guarantees granted either by EGAP (Exportní garanční a pojišťovací společnost, a.s.) or by the National Development Bank, principally in support of SMEs. These programmes were primarily intended to facilitate access to operational financing following the economic consequences caused by the measures taken to counter the spread of Covid-19, especially if businesses were forced to reduce or stop their operations due to these measures.^16^ Later, indirect financial support was also available for certain large companies (the Covid Plus programme was used, for instance, by Smartwings airlines, which entered into a loan agreement with a syndicate of banks in March 2021 for CZK 2 billion that was guaranteed by EGAP).

## Lex Covid and Corporate Governance

In response to the restrictions outlined above and the problems associated with the inability of (especially) business corporations to hold meetings in person, the Czech ‘Lex Covid’^17^ was adopted in an accelerated procedure in order to introduce special corporate governance rules for business corporations,^18^ with effect from 24 April 2020, which would not apply under normal circumstances. These measures were introduced in addition to other measures to respond to the free movement restrictions and other adverse consequences resulting from the pandemic, whether in the area of corporate governance, litigation, insolvency (see below), or enforcement of decisions. The statutory measures were to apply during a transitional period which would equal the duration of the emergency measures, but only until 31 December 2020, unless Lex Covid provided otherwise. Due to the duration of the pandemic, certain relief and mitigation measures in the area of justice, insolvency and enforcement of decisions were subsequently extended (so-called Lex Covid II^19^). The amendment extended the effects of certain measures to mitigate complications similar to those arising from the pandemic wave in the autumn of 2020.

In the area of corporate governance, Lex Covid’s aim was to ensure that decision-making within business corporations would not be completely paralysed during the emergency situation and that the corporations could continue to operate. To that end, Lex Covid (i) prolonged the term of service for elected body members whose term was set to expire during the emergency measures or within 1 month of the day following the expiry of the emergency measures, which restricted or could have restricted the possibility to hold a general meeting to elect new board members; and (ii) allowed, for as long as the emergency measures applied (but no later than 31 December 2020), any body of a legal entity (in particular the general meeting or members’ meetings of business corporations, including companies whose participating securities were admitted to trading on a regulated market) to take decisions outside a regular meeting (*per rollam*) or by using electronic devices, even if this was not allowed according to the particular business corporation’s memorandum of association or articles of association.

Despite the initial shock, Lex Covid allowed Czech companies to function properly in terms of corporate governance, e.g., to appoint new members of the statutory bodies (crisis managers) *per rollam* even if the statutes did not allow such procedure, or to approve additional equity funding by the respective corporate bodies. Hence, potential obstacles in terms of corporate governance laws were removed. What remained were potential financial problems of Czech companies resulting from the pandemic and subsequently introduced emergency measures.

## Lex Covid and Insolvency—Restructuring Laws

Given the anticipated problems for businesses caused by the drop in income and liquidity as a result of the pandemic, special legislation on insolvencies and restructurings was adopted as early as the end of April 2020, with the intention of enabling troubled businesses, in particular those in insolvency but still economically viable, to manage their difficult situation with the help of special instruments that could be used in or in the context of insolvency proceedings. The government considered the standard regulatory framework to be institutionally insufficient, and, responding to public demand, adopted a set of temporary regulatory measures to relieve entrepreneurs in trouble. The main statutory changes were the following:

(i) *Creditor insolvency petitions were inadmissible*

Lex Covid’s aim was, among other things, to temporarily protect businesses from entering formal insolvency proceedings, whether on a voluntary or involuntary basis. First of all, for a transitional period ending on 31 August 2020, creditors were *ex lege* stripped of the opportunity to initiate insolvency proceedings against their debtors by filing an insolvency petition. The purpose of this measure was to give debtors ‘breathing room’ to ensure that their operations continued on a going concern basis during the transitional period.

The law excluded the right of a creditor to initiate insolvency proceedings against the debtor for both (a) *insolvency* (based on a test according to which debtors are considered insolvent when having multiple creditors, monetary obligations for a period longer than 30 days after the due date which they are not able to fulfil), and (b) *over-indebtedness* (under which test debtors are considered insolvent if they have multiple creditors and their aggregate liabilities exceed the value of their assets). Similarly, it was legally irrelevant whether the debtor’s insolvency occurred before or after the anti-Covid restrictive measures were adopted, or what the reason for the debtor’s insolvency was. The Czech Parliament adopted a catch-all principle as creditors were temporarily unable to file an insolvency petition even if the reason for the debtor’s insolvency was not linked to the Covid-19 pandemic or the emergency measures adopted. The only factor that was important was the moment the creditors’ insolvency petition was filed.

If the insolvency petition was filed (delivered to the insolvency court) between 24 April 2020 and 31 August 2020, it was completely disregarded, i.e., it did not trigger any ‘material’ effects (e.g., it did not initiate the limitation and prescription periods for rights that could only be exercised by way of an application if insolvency proceedings were initiated) or ‘procedural’ effects (in particular, it did not trigger insolvency proceedings). Therefore, for example, the filing of a creditor’s insolvency petition did not prevent the creditor from asserting claims or other rights against the debtor’s assets before a civil court, and (with exceptions) enforcement of judgments could continue to be carried out if not blocked by other measures. However, as blocking instruments (such as an extraordinary moratorium) were rarely used to stop unilateral enforcement actions (see below), one can make the argument that the governmental financial aid programmes enabled businesses largely to continue honouring fixed obligations or, at least, arranging g standstill arrangements with their creditors during the crisis period. On the other hand, since creditors were unable to file a petition to initiate insolvency proceedings, they were generally unable to restrict the debtor in disposing of its assets whilst the debtor was not even required to file a debtor’s insolvency petition unless it was over-indebted (see below). As the temporary measure was designed to avoid insolvency proceedings, Lex Covid extended the deadlines by which the debtor’s conduct could be challenged by an insolvency trustee if one was to be appointed later when the temporary restrictions were lifted.

(ii) *The debtor’s duty to file an insolvency petition was suspended*

The disproportionate concentration of losses in various sectors of the Czech economy would create a risk of fire-sale outcomes should a substantial part of the economy commence a collective insolvency procedure.^20^ Therefore, Lex Covid stipulated that the obligation of a debtor to file an insolvency petition was suspended for a transitional period from the date Lex Covid became enforceable until the expiry of 6 months after the epidemic emergency measure was terminated or revoked, but no later than until 31 December 2020. The law was based on the premise that, as a result of the adoption of the emergency measures prohibiting/restricting certain forms of businesses, affected businesses might become temporarily insolvent, yet such financial difficulties might be overcome once the emergency measures were lifted. This did not exclude the debtor’s option to file an insolvency petition voluntarily. Nevertheless, no negative legal consequences were attached to the failure to file an insolvency petition if the conditions for insolvency were met for the duration of this exception.

However, this rule generally did not apply to:

- entities whose insolvency occurred before the adoption of the emergency measures (i.e., realistically, before 12 March 2020). Insofar as a debtor went bankrupt independently of the Covid-19 pandemic, Czech lawmakers did not consider it necessary to grant debtors special protection the purpose of which was in particular to overcome the negative effects of the epidemic in terms of fulfilment of monetary obligations; or
- cases in which the state of insolvency was not caused (primarily) by emergency measures prohibiting/restricting certain forms of businesses. The burden of judgment was on debtors, which were required to consider the impact of the extraordinary legislative measures on their business. If the extraordinary measures (prohibiting/restricting certain forms of businesses) adopted by the government during the pandemic only contributed to, or accelerated, the debtor’s bankruptcy, but were not the predominant reason and main cause (i.e., bankruptcy would have occurred independently of the extraordinary measures adopted during the pandemic), debtors would still be required to file an insolvency petition.

Czech law provides for two forms of bankruptcy: (a) insolvency, and (b) over-indebtedness (see the definitions above). Czech legislators suspended the debtor’s duty to file an insolvency petition where the debtor’s bankruptcy was predominantly caused by emergency legislative measures (prohibiting/restricting certain forms of businesses) that ‘would prevent or substantially impede the debtor from meeting its monetary obligations’.^21^ Failure to meet (due) monetary obligations relates to insolvency, not to over-indebtedness. Therefore, the debtor’s duty to file an insolvency petition was supposedly suspended only if the debtor became insolvent during the transitional period, not if it became over-indebted. In such a case, the debtor’s obligation to file an insolvency petition remained unaffected.^22^ We did not see, however, any increased number of debtor insolvency filings based on indebtedness during this period, which may suggest that the governmental financial aid measures were rather generous. In addition, as it is difficult to determine over-indebtedness in ordinary times, it becomes a nightmare to do so during a crisis, given valuation uncertainty (market disruption, temporary illiquidity of the market, etc.).

Since the debtor was not required to file an insolvency petition if it became insolvent during the interim period, this also automatically suspended the obligation of the debtor’s statutory representatives to file an insolvency petition. According to ‘standard’ insolvency regulation,^23^ a company’s executive body must file an insolvency petition *without undue delay* after it becomes aware or, with due diligence, should have become aware of its insolvency. Otherwise, members of the company’s executive body are liable to creditors for the damage caused by the late filing of the insolvency petition.^24^ Not only did the debtor’s management thus gain room to find a commercial solution, but also, and in particular, members of the debtor’s statutory bodies gained protection in terms of liability for failure to initiate insolvency proceedings in a timely manner.

Under Lex Covid II, the debtor’s obligation to file for insolvency was suspended even until 30 June 2021. However, the measure that restricted creditors from filing creditor insolvency petitions from 17 April 2020 to 31 August 2020 was not renewed under Lex Covid II.

(iii) *Special measures related to the implementation of a reorganisation plan*

Lex Covid also allowed debtors to temporarily suspend the implementation of a reorganisation plan without the threat of the reorganisation turning into bankruptcy. The relief applied to insolvency proceedings in which a debtor’s reorganisation plan was finally approved by 12 March 2020 but had not yet been fully implemented, regardless of who submitted the plan (whether the debtor or the creditors).^25^

The debtor was entitled to file a motion requesting the insolvency court to decide that the debtor was entitled to temporarily suspend the implementation of the reorganisation plan, at the latest until the end of the period during which the effects of the emergency measures lasted (i.e., the same period during which it was not effectively possible to file a creditor’s insolvency petition).^26^ If the motion was granted, the ongoing reorganisation could not be converted into bankruptcy on the grounds of the debtor’s failure to meet the conditions and covenants set out in the reorganisation plan during the period of the suspension (i.e., if the debtor stopped fulfilling its historical debts included in the reorganisation plan in the manner and by the deadlines foreseen in the plan).

On the other hand, Lex Covid did not limit the debtor’s obligation to perform obligations other than those included in the approved reorganisation plan. For those cases, the law did not explicitly exclude the possibility of converting the reorganisation into bankruptcy on the grounds of non-performance of ‘other outstanding monetary obligations’ within the meaning of the Insolvency Act. The suspension of the debtor’s obligation to fulfil its obligations under the approved reorganisation plan was without prejudice to other parties’ obligations (for example, creditors or business partners) to fulfil their obligations towards the debtor.

(iv) *Extraordinary (temporary) moratorium*

When Czech businesses were mainly struggling with the impact of the Covid-19 epidemic between 2020 and 2021, EU Directive 2019/1023 on restructuring and insolvency was not implemented in the Czech Republic.^27^ Therefore, Czech businesses were unable to use the tools that preventive restructuring in the sense of this Directive could have brought. However, Lex Covid introduced a so-called extraordinary moratorium into the Insolvency Act (Section 127a).^28^

The purpose of an extraordinary moratorium was to provide debtors who ran into temporary problems in connection with the Covid-19 pandemic with an instrument of protection against individual creditors (preventively before or after the insolvency petition was filed, but always before the decision on the insolvency petition was made) and thus to provide debtors with additional time to solve their financial difficulties, i.e., to overcome, or at least effectively address, the shortfall in available funds. In general, an extraordinary moratorium sought to avoid fire-sale outcomes should a substantial part of the economy enter formal insolvency proceedings at the same time. Company reorganisations, which generally avoid fire-sales of the debtor’s assets, would not serve this purpose properly at a time of a crisis such as the one created by the Covid-19 pandemic. Most Covid-distressed firms did not require economic or financial restructuring within the scope of insolvency law. Rather, they experienced massive, but temporary, cash-flow problems as their revenues basically evaporated overnight due to the introduced emergency measures.^29^

The extraordinary moratorium was therefore designed to provide temporary protection from creditors’ individual courses of action (breathing room). An application for an extraordinary moratorium could be filed outside insolvency proceedings, i.e., without an insolvency petition being filed simultaneously (either by the debtor or, in the previous period, by the creditor). From this perspective, the extraordinary moratorium had a preventive function as it was able to help to avert impending insolvency or negotiate a restructuring solution with creditors.

The formalities of the application for the extraordinary moratorium were relatively loosely defined compared to the application for a ‘standard’ moratorium (for example, the debtor did not have to provide a list of liabilities or an inventory of assets). The requirements under which the court could declare an extraordinary moratorium were as follows:^30^

(i) The debtor was not bankrupt prior to 12 March 2020 (in the form of insolvency or over-indebtedness);
(ii) The debtor’s centre of main interests (COMI) was in the Czech Republic;
(iii) The extraordinary moratorium was proposed as a result of emergency measures or other measures taken by the Czech Republic in response to the spread of the Covid-19 disease caused by the new SARS CoV-2 coronavirus;
(iv) The debtor did not, during the 2 months prior to 12 March 2020 or thereafter, pay extraordinary profit shares to or otherwise distribute equity to, or provide other extraordinary benefits to, its members, partners or shareholders or parties controlling or controlled by them or members of their bodies, including early repayment of loans or borrowings, unless all benefits so provided were repaid.

The extraordinary moratorium conditions foresaw that, in the first few months, profit shares in Czech companies could be paid out in full compliance with the law, at a time when no one could have expected such a widespread coronavirus epidemic. However, since the extraordinary moratorium could affect creditors’ rights, this condition was considered a compromise between the debtor’s and creditors’ interests in that companies which had decided to pay out profit shares or other distributable funds between 12 January to 12 March could apply for an extraordinary moratorium, provided that all such payments were repaid back to the debtor. However, the law did not require members, partners or shareholders, or members of elected bodies or other affiliated parties which received such payments to repay the consideration back to the company. This would have to be done on a voluntary basis, which, especially in companies with a larger number of members or shareholders, could be a Herculean task, as the law required that 100% of the profit or share of other distributable funds paid out should be returned in this way. On the other hand, practically speaking, it is uncommon for companies to pay out shares in profits or other distributable funds in the first months of the year if their accounting period corresponds to the calendar year, since the first months of the year are usually devoted to preparing the financial statements for the previous accounting period, which form the basis for the subsequent decision on distribution of profits or other distributable funds.

Reviews of the statutory conditions to apply for an extraordinary moratorium (such as non-existence of bankruptcy prior to 12 March 2020) were relatively lenient. The court review was basically a formality. All the debtor had to do was to prepare and submit an affidavit. A significant change from the standard moratorium provided for in the Insolvency Act was the waiver of the requirement of creditors’ prior consent for declaring an extraordinary moratorium, which was intended to ensure in particular the operability of the measure and the possibility of its rapid application.^31^ A grace period of 3 months was supposed to be granted to basically anybody who made a request. If the debtor failed to resolve its financial problems during the first grace period, the protection of creditors under the conditions of the ‘standard’ moratorium was reintroduced. Hence, the extraordinary moratorium could be extended for an additional three months; however, the creditors’ consent to the extension had to be secured here.

During an extraordinary moratorium, the court could not issue a decision on the debtor’s insolvency or enforce a claim against the debtor. Therefore, for example, if any of the debtor’s creditors filed a creditor’s petition before 12 March 2020 (i.e., before the date of the statutory suspension of creditors’ authority to file a creditor’s petition), the debtor could prevent the declaration of bankruptcy by filing a petition for an extraordinary moratorium. If the extraordinary moratorium was granted, the debtor would be authorised to pay debts directly related to preserving the debtor’s business that became due after the declaration of the extraordinary moratorium prior to previously due debts. Additionally, contracts for the supply of energy and raw materials, as well as other contracts for the supply of goods and services which had been in force for at least 3 months at the date of the declaration of the extraordinary moratorium, could not be terminated or withdrawn by the debtor’s counterparty during the period of the moratorium (Sections 127a (6) and 122 (2) of the Insolvency Act).

An extraordinary moratorium would, in many cases, be insufficient in terms of saving the debtor’s business during the restrictive measures if, at the same time, the debtor could not benefit from the public support programmes (state, EU or local government) introduced during the Covid-19 pandemic. Therefore, Lex Covid expressly stipulated that businesses that applied for the extraordinary moratorium would also be able to apply for the public support and that there were no negative consequences associated with using it in terms of the duration of the extraordinary moratorium. On the other hand, the extraordinary moratorium also entailed certain obligations and restrictions for the debtor, in particular as regards disposal of assets. The debtor was obliged to refrain from disposing of the estate and any assets that might belong to it if there were to be substantial changes in the composition, use or destination of those assets or a not insignificant reduction in their size.^32^

## What Happened Next?

**Fig. 1:**
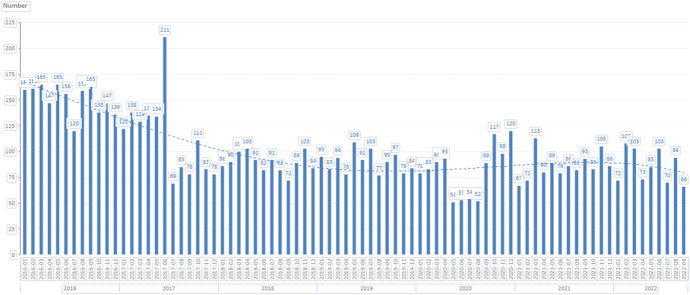
Number of insolvency petitions filed in the Czech Republic by month since 1 January 2016. Source: Surveilligence, s.r.o. (https://www.surveilligence.com/sk)

Lex Covid, both I and II, brought significant relief to domestic businesses which had experienced temporary economic difficulties as a result of the Covid-19 pandemic and subsequently introduced extraordinary measures. However, the protective measures had their limitations in terms of duration. For instance, with effect from 31 August 2020, the right of creditors to file insolvency petitions against debtors was restored. However, the generally expected increase in insolvency filings did not occur. The number of insolvency petitions filed after the end of the transitional period was, according to some, ‘suspiciously low’.^33^ For example, according to public data, in the first half of September 2020, only approximately 49 creditor insolvency petitions were filed (by comparison, there were only 20 fewer in the same period in 2019), while the total number of insolvency petitions (both creditors’ and debtors’ insolvency petitions) was basically the same in September 2020 as in September 2019 (89 and 90, respectively; see chart below). Surprisingly, the number of newly initiated insolvency proceedings in the Czech Republic in 2020 was lower than in any given year between 2008 and 2019 (see Fig. 1).^34^

One would expect that structural difficulties of part of the Czech economy would manifest themselves with some delay. Even by the end of 2021, however, we did not see any significant increase in the number of insolvency proceedings in the Czech Republic. According to data analysis company InsolCentrum, the number of bankruptcies rose by 18% in 2021 in year-to-year comparison.^35^ However, in 2020, only 612 bankruptcies were declared (the lowest number in the past 10 years). Actually, the number of bankruptcies declared in 2021 was the 4^th^ lowest in the last 10 years. Undoubtedly, state subsidy programmes, which were described by entrepreneurs and the public as relatively generous, played a significant role, as did the fact that interest-free loans which were provided to Czech businesses under the Covid-19 financial aid programmes were not repayable by the end of 2021.

**Fig. 2:**
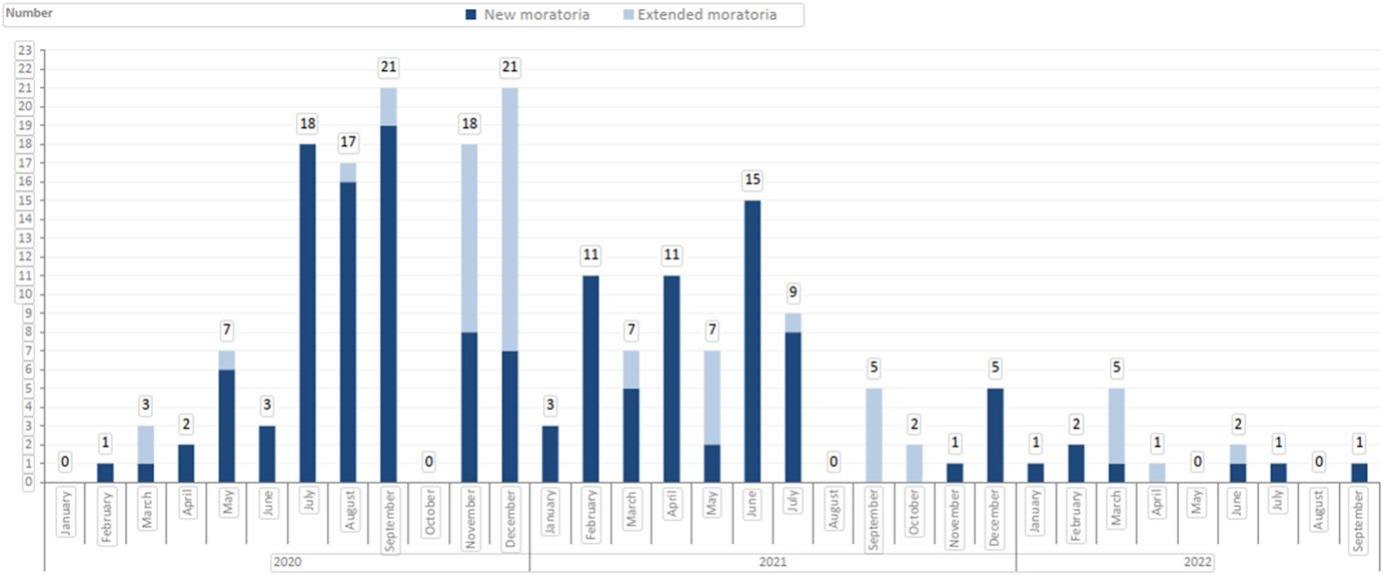
Number of moratoria in the Czech Republic by month since 1 January 2020. Source: Surveilligence, s.r.o. (https://www.surveilligence.com/sk)

The existence of state bail-out programmes seems to have had an impact also on the extent to which Czech businesses took advantage of the extraordinary moratorium. Although relatively lenient requirements were set for declaring an extraordinary moratorium, slightly more than 40 companies (e.g., FIRO-tour, Smartwings and Czech Airlines)^36^ applied to the court for an extraordinary moratorium (within the first phase, i.e., until the end of August 2020), and approximately the same number of companies applied for an extraordinary moratorium during the second phase (until 30 June 2021; see chart below). Although, according to the explanatory memorandum to Lex Covid II, the extraordinary moratorium has proved its worth, the number of petitions for an extraordinary moratorium was clearly not as high as expected, which may have been caused, again, by the combination of relatively generous state subsidy programmes, the prohibition for creditors to file creditor insolvency petitions, and/or the possibility of deferring loan repayments subject to the requirements imposed by Act No. 177/2020 Coll (see Fig. 2).

Since creditor insolvency petitions did not rise in any appreciable way following the end of the restriction for creditors to file creditor insolvency petitions, and petitions for an extraordinary moratorium did not increase in any significant way after 31 August 2020, one may assume that the state bail-out programmes were relatively pinpointed in terms of rescuing the most affected entities. As the number of insolvency petitions was actually less in comparison to previous years, one may even argue that these financial aid programmes were too generous. If governmental bail-out programmes are generous enough, insolvency law becomes largely irrelevant during crises.

Yet, I recall walking through the city of Prague and other Czech cities after 2020 and seeing so many shops and restaurants shut down. Many of these (rather small) businesses likely just voluntarily closed down and were liquidated as a result of the pandemic, which had a material negative effect on them without forcing them to enter into insolvency proceedings. At the same time, from a practical point of view, we have seen cases of private transfers of distressed assets within the distressed M&A market. In contrast to 2020, when M&A activity declined noticeably year-on-year, we noticed an upbeat note in the Czech M&A market in 2021 with a similar number of transactions as in the pre-pandemic period including an increased number of so-called sell-side transactions. According to KPMG, ‘performance in the mergers and acquisitions (M&A) market in the Czech Republic and Slovakia in 2021 was one of the record highs in terms of number and volume of transactions’,^37^ with this market seeing the most activity in the real estate/construction sector, which was hit hard during the pandemic,^38^ and in the e-commerce area in 2021. Although distressed M&A was an important part of the M&A market after the pandemic, general expectations regarding distressed sales as the main driver of the entire M&A market have not materialised.^39^ This would also suggest that, due to the generous bail-out programmes, not many sellers were, contrary to general expectations, forced to offer their businesses for sale.

There’s no such thing as a free lunch, though. The public subsidy programmes adopted in the Czech Republic in response to the Covid-19 pandemic significantly increased public debt, and some commentators have even suggested that the Czech Republic is on the ‘Greek path’. In addition, ‘helicopter money’ provided under the financial aid programmes (such as the compensation bonus and Antivirus) and savings by the general public, which had limited ways of being spent during the pandemic (travels abroad as well as ‘off-line’ shopping were limited), found their way into the economy when the emergency measures were lifted and, in addition to increased energy prices, triggered a significant increase in the inflation rate in the Czech Republic.^40^

## Summary

In the course of the Covid-19 global pandemic, corporate restructuring laws underwent a stress test as the financial health of companies significantly deteriorated. This paper outlined the basic framework of the measures adopted in the Czech Republic in response to the crisis. On the bright side, Covid-19 has sped up processes of digitalisation and automation in various business (e.g., in the banking and insurance industry). Many processes were transferred to the online world, and online meetings have become an integral part of the economy.

In response to public demand, the Czech legislators adopted various temporary measures to mitigate the impact of the Covid-19 pandemic. In addition to several state aid programmes, various measures were introduced in relation to corporate restructuring laws, e.g., for a transitional period, creditors were stripped of the opportunity to initiate insolvency proceedings by filing an insolvency petition against their debtor, the obligation of a debtor to file an insolvency petition was suspended, and an extraordinary moratorium was introduced in order to provide debtors that ran into temporary problems in connection with the Covid-19 pandemic with protection against individual creditors and to solve their financial difficulties. However, the generally expected increase in insolvency proceedings has (so far) not occurred after the pandemic, while the increased wave of company insolvencies taking place at the end of 2022 is rather related to the energy crisis resulting from the Russian invasion of Ukraine.

As the number of insolvency petitions actually decreased after the pandemic in comparison to previous years and, contrary to general expectations, distressed sales have (so far) not become the main driver of the entire M&A market in the Czech Republic, one may argue that these financial aid programmes were even too generous in the Czech Republic. As a result, however, Czech public debt has increased dramatically while ‘helicopter money’ provided under the governmental bail-out/financial aid programmes has significantly accelerated the increase in the inflation rate in the Czech Republic. And the impacts of the ongoing energy crisis are already on our doorstep.

## Footnotes

1 Certain emergency measures adopted by the Ministry of Health were abolished by a judgment of the Municipal Court in Prague of 23 April 2020 on the grounds that the Ministry failed to have jurisdiction to issue such measures; only the government could do so. Therefore, on 23 April 2020, the government issued new emergency measure No. 452, which, as of 27 April 2020 until the end of the state of emergency (until 17 May 2020), amended the conditions for the movement of individuals in the Czech Republic.

2 Act No. 240/2000 Coll., as amended.

3 These legal as well as factual limitations obviously jeopardised how businesses in the Czech Republic operated. Without effective decision-making of general meetings or other bodies authorised to elect members of the statutory body being ensured, there were situations, especially in companies with more than a few members/shareholders, where the existing directors (members of the executive body) ceased to serve and it was not legally possible (or, given the public health regulations, permissible) to elect new members. In such cases, companies could find themselves without a body managing their operations and/or authorised signatories, i.e., they may have been completely paralysed while dealing with severe financial cash-flow problems resulting from the pandemic.

4 Cf. van Zwieten et al. (2021), pp 201–202.

5 Communication from the Commission: Temporary Framework for State aid measures to support the economy in the current Covid-19 outbreak (as adopted on 19 March 2020 (C(2020) 1863) and its amendments C(2020) 2215 of 3 April 2020, C(2020) 3156 of 8 May 2020, C(2020) 4509 of 29 June 2020, C(2020) 7127 of 13 October 2020, C(2021) 564 of 28 January 2021, and C(2021) 8442 of 18 November 2021).

6 On the differences between bail-in (mandating some forgiveness by creditors and/or counterparties in the form of, e.g., interest write-down, mandatory extension of a debt’s maturity or temporary moratorium) and bail-out measures (borne by taxpayers over time, such as state cash grants, state loans or loan guarantees), see generally Zhou et al. (2012), or van Zwieten et al. (2021), p 510.

7 On 27 March 2020—with promulgation in the Federal Law Gazette—German legislators introduced a law ‘to mitigate the consequences of the Covid-19 pandemic in civil, bankruptcy and criminal procedure law’ which included a temporary suspension of landlords’ termination right in the case of Covid-19-caused payment defaults occurring from 1 April to 30 June 2020 that applied to both residential and commercial leases.

8 The protection of tenants was reflected in Czech Acts No. 209/2020 Coll. and 210/2020 Coll.

9 See the Ministry of Industry and Trade’s website: Covid-Rents programme, https://www.mpo.cz/podnikani/zivnostenske-podnikani/Covid-19-najemne—255305 (accessed 16 December 2022).

10 The Czech Banking Association also called on banks to allow borrowers to suspend loan repayments if they lost their ability to repay their loans in connection with the pandemic.

11 See Section 29 of Act No. 191/2020 Coll., on certain measures to mitigate the effects of the SARS CoV-2 coronavirus epidemic on parties participating in court proceedings, aggrieved parties, victims of crime and legal entities, and on the amendment of the Insolvency Act and the Code of Civil Procedure (coined ‘Lex Covid’ in the Czech Republic).

12 See Ondrejová (2021), p 2 et seq.

13 See the Ministry of Industry and Trade’s website: Covid programmes for companies and entrepreneurs, https://www.mpo.cz/cz/rozcestnik/informace-o-koronavirus/Covid-programy-pro-firmy-a-osvc—259263 (accessed 16 December 2022).

14 The compensation bonus was designed to provide financial support primarily to small businesses (i.e., individual entrepreneurs or members of limited liability companies) which incurred a reduction in revenues of more than 10% and (after October 2020) of more than 30%. Recipients under the subsidy programme were entitled to receive approx. CZK 15,000 (approx. EUR 630) per month until 31 January 2021 and approx. CZK 30,000 (approx. EUR 1260) for the period between 1 February and 31 May 2021. In spring 2021, the Ministry of Finance reported around 1 million requests and in autumn 2021 approx. 835,000). More than CZK 44 billion (slightly less than EUR 2 billion) was paid out under the compensation bonus programme.

15 Businesses whose predominant activities were affected by government prohibitions or restrictions had all VAT payments automatically deferred until the end of the year and advance payments of income tax and road tax waived.

16 For example, under the Covid I programme, businesses were entitled to receive an interest-free loan of up to EUR 15 million for up to 90% of eligible project costs, repayable over up to 2 years with a 12-month grace period. Under the ‘Covid III invest’ or ‘Covid III provoz’ programmes, on the other hand, businesses with up to 500 employees could apply for support in the form of a guarantee, which was graded according to the number of employees and the form of the loan (operating vs. investment).

17 Act No. 191/2020 Coll., on certain measures to mitigate the effects of the SARS CoV-2 coronavirus epidemic on parties participating in court proceedings, aggrieved parties, victims of crime and legal entities, and on the amendment of the Insolvency Act and the Code of Civil Procedure.

18 For the purposes of Czech law, a business corporation is deemed to mean a public business company, a limited partnership, a limited liability company, a joint-stock company or a cooperative.

19 Act No. 460/2020 Coll., amending the Act on certain measures to mitigate the effects of the SARS CoV-2 coronavirus epidemic on parties participating in court proceedings, aggrieved parties, victims of crime and legal entities and on the amendment of the Insolvency Act and the Code of Civil Procedure (coined ‘Lex Covid II' in the Czech Republic).

20 Which may be detrimental to both debtors and their creditors. Cf. van Zwieten et al. (2021), p 213.

21 Section 12 (1) of Lex Covid.

22 See Zezulka (2020).

23 Section 98 of the Insolvency Act.

24 Section 99 of the Insolvency Act.

25 Based on Lex Covid II, the possibility to suspend the implementation of the reorganisation plan due to the anti-epidemic measures was extended until 30 June 2021.

26 The decision required the approval of both the insolvency administrator and, in particular, the creditors’ committee. No appeals against the decision were admissible. However, the insolvency court could take measures to protect the legitimate interests of creditors, if necessary.

27 In January 2022, a draft law on preventive restructuring was submitted to the Czech government for consideration which aims to transpose Directive 2019/1023 on restructuring and insolvency. Preventive restructuring is supposed to represent a new legal institute intended for entrepreneurial legal entities in temporary financial difficulties.

28 For a most detailed analysis of extraordinary moratorium regulation introduced into Czech law during the pandemic, see Sprinz (2020), p 343 et seq.

29 Cf. Eidenmüller and Valbuena (2021), p 508.

30 Section 127a (2) of the Insolvency Act as amended by Lex Covid.

31 See Sprinz (2020), p 344.

32 Based on Lex Covid II, the debtor’s opportunity to apply for an extraordinary moratorium under Section 127a of the Insolvency Act was extended until 30 June 2021. Debtors who had already benefited from the extraordinary moratorium, however, could no longer take advantage of it. Even in that case, the suspension of the debtor’s obligation to file an insolvency petition against itself (the debtor) still applied until 30 June 2021.

33 Cf. interview with Tomáš Valášek from Insolvence 2008, reprinted in Hlaváčková (2020), p 14.

34 See also IsirExplorer (2021).

35 See CzechInvest (2022).

36 See Hlaváčková (2020), p 15.

37 See Kantor (2022).

38 See InsolCentrum (2021).

39 See KPMG (2022).

40 The Czech Republic currently has one of the highest inflation rates in the EU. According to Eurostat, the annual inflation rate in the Czech Republic was 17.8% in September 2022, while the euro area annual inflation rate was 9.9%. See Eurostat (2022).
